# Lead Halide Perovskite Photoelectrocatalysis

**DOI:** 10.1002/adma.73442

**Published:** 2026-05-23

**Authors:** Virgil Andrei

**Affiliations:** ^1^ School of Materials Science and Engineering Nanyang Technological University Singapore Singapore; ^2^ Energy Research Institute at Nanyang Technological University (ERI@N) Singapore Singapore; ^3^ Yusuf Hamied Department of Chemistry University of Cambridge Cambridge United Kingdom

## Abstract

Lead halide perovskites have emerged as an outstanding class of light harvesting materials. While much progress has been made in solar cell efficiency, the materials also proved potential for light‐driven conversion of water, CO_2_ or organic waste streams into solar fuels and value‐added chemicals. This case study highlights advances made in the rational photoelectrode design to improve solar‐to‐chemical conversion efficiency, product scope, and scalability. To this end, we will explore the interplay between device architecture and performance, strategies to suppress moisture degradation pathways, as well as fundamental mechanisms to steer selectivity of CO_2_ reduction products. These insights are generalizable to a wide scope of thin‐film buried‐junction photoelectrodes, highlighting the unique applicability of this technology to real‐world scenarios.

## Graphical abstract

Lead halide perovskite semiconductors have emerged as highly promising materials for solar fuel and chemical synthesis. This perspective discusses advances made in the rational photoelectrode design to improve solar‐to‐chemical conversion, product scope, and scalability. To this end, we will explore the interplay between device architecture and performance, moisture degradation pathways and mechanisms to steer product selectivity.

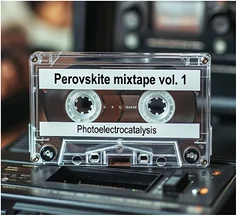

## 1 Introduction

**FIGURE 1:**
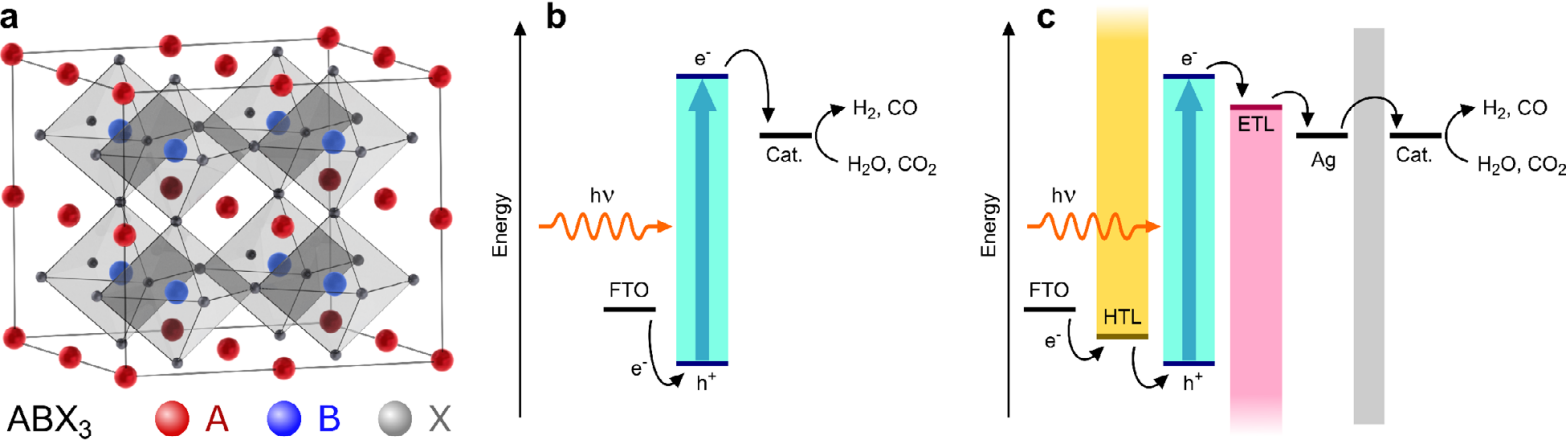
Fundamental design of perovskite photoelectrodes. (a) The general ABX_3_ perovskite crystal structure displays BX_6_ octahedra and a cuboctahedral coordination of the A cation. (b) Classical photocathode with a semiconductor‐electrolyte interface and surface bound reduction catalyst. (c) Buried‐junction perovskite photocathode. The semiconductor is sandwiched between charge‐selective layers, while a conductive moisture barrier enables charge transfer to a surface catalyst.

Perovskites are a class of materials possessing an ABX_3_ chemical formula, where A and B are cations of different sizes, and X is the anion. In this structure, the B cations are surrounded by six X anions in an octahedral coordination. The A cations are located between BX_6_ octahedra, in a 12‐fold cuboctahedral coordination [1]. A depiction of the perovskite lattice comprising of eight crystal unit cells is given in Figure 1a. To form the perovskite structure, the ionic radii *R* need to abide to the relationship $R_{A}+R_{B}=t\;\times \;\sqrt{2}\left(R_{B}+R_{X}\right)$, where the tolerance factor *t* typically varies between 0.8 and 1. Traditionally, this class of materials corresponds to oxides with the crystal structure of the mineral CaTiO_3_. Over the past couple decades, lead halide perovskites have emerged as a highly promising class of semiconductors. While initial reports established perovskite light absorbers for photovoltaic (PV) devices [2, 3, 4], these soon found applications for solar fuel synthesis, either in PV‐electrolysers [5], as photocatalyst nanoparticles [6, 7], or in buried‐junction photoelectrodes. Here, we will focus on the progress made in photoelectrode development, highlighting their unique design features among solar fuel technologies.

## 2 Photoelectrode Architecture

### 2.1 Design Versus Functionality

Historically, photoelectrocatalytic (PEC) systems have been defined by the presence of a semiconductor‐electrolyte interface (Figure 1b), which would induce band‐bending effects promoting charge separation. For these classical photoelectrodes, functionality was heavily influenced by the *p*‐ or *n*‐type character of the semiconductor and its energy levels, which would favor either reductive or oxidative reactions (i.e., photocathodes or photoanodes) [8]. However, due to either insufficient charge extraction, voltage losses, high catalytic overpotentials, surface recombination or photocorrosion, these electrodes would display low photocurrents and a moderate stability. To overcome these challenges, efforts have gradually shifted from interfacing semiconductors with selective electrocatalysts (Figure 1b) [9, 10], to introducing moisture protective barriers (e.g., TiO_2_, ITO) [11], and designing heterojunctions with carefully aligned energy levels [12, 13]. Such insights in designing device architectures have been well known to the photovoltaic (PV) community for decades, and proved indeed highly beneficial in inspiring the PEC community.

In contrast to classical photoelectrode materials, metal halide perovskites are intrinsic semiconductors and highly moisture sensitive [1]. Therefore, their integration in solution‐immersed photoelectrodes requires both electron and hole transport layers (ETL/HTL) for selective charge separation [1], as well as an effective moisture barrier conducting charges to the surface catalyst (Figure 1c) [14]. While these so‐called buried‐junction or buried‐PV photoelectrodes can benefit from insights in both (photo)electrocatalysis and photovoltaics, they can also raise questions.

*‘What is a photoelectrode?’* Coming mostly from the photoelectrocatalysis community, this general question raises the argument whether the buried‐heterojunction electrode is indeed comparable to a classical photoelectrode. As band bending and photodegradation are reduced, the operating principle deviates from that of a ‘true’ photoelectrode. Additional layers may also add complexity or material cost to the devices, taking away the balance‐of‐cost advantage of classical PEC systems compared to PV‐electrolysis [15].

*‘How thick is the wire?’* Originating mainly from the PV‐electrolysis community, this viewpoint questions whether the perovskite integration in submerged electrodes is even necessary. While the thin‐film PV community possesses well‐established protocols for device fabrication at scale, questions are raised whether a spatial decoupling of light absorption and catalysis is not in fact beneficial for overall solar‐to‐fuel conversion efficiency (STF) and stability. From this angle, the conductive moisture barrier is only a very short wire, hence an integrated system would not reveal any fundamental insights compared to ongoing photovoltaic and electrocatalysis research [16].

To answer these questions, this perspective will highlight some unique features and benefits of buried‐junction photoelectrocatalytic systems, focusing on metal halide perovskite photoelectrodes as a case study. Here, we make the argument that functionality, rather than design, is what ultimately defines a photoelectrode [17]. That is, a photoelectrode must combine its two basic functions of light harvesting (i.e., *photo*) and electrocatalysis.

### 2.2 Rational Design of Charge Selective Layers

**FIGURE 2:**
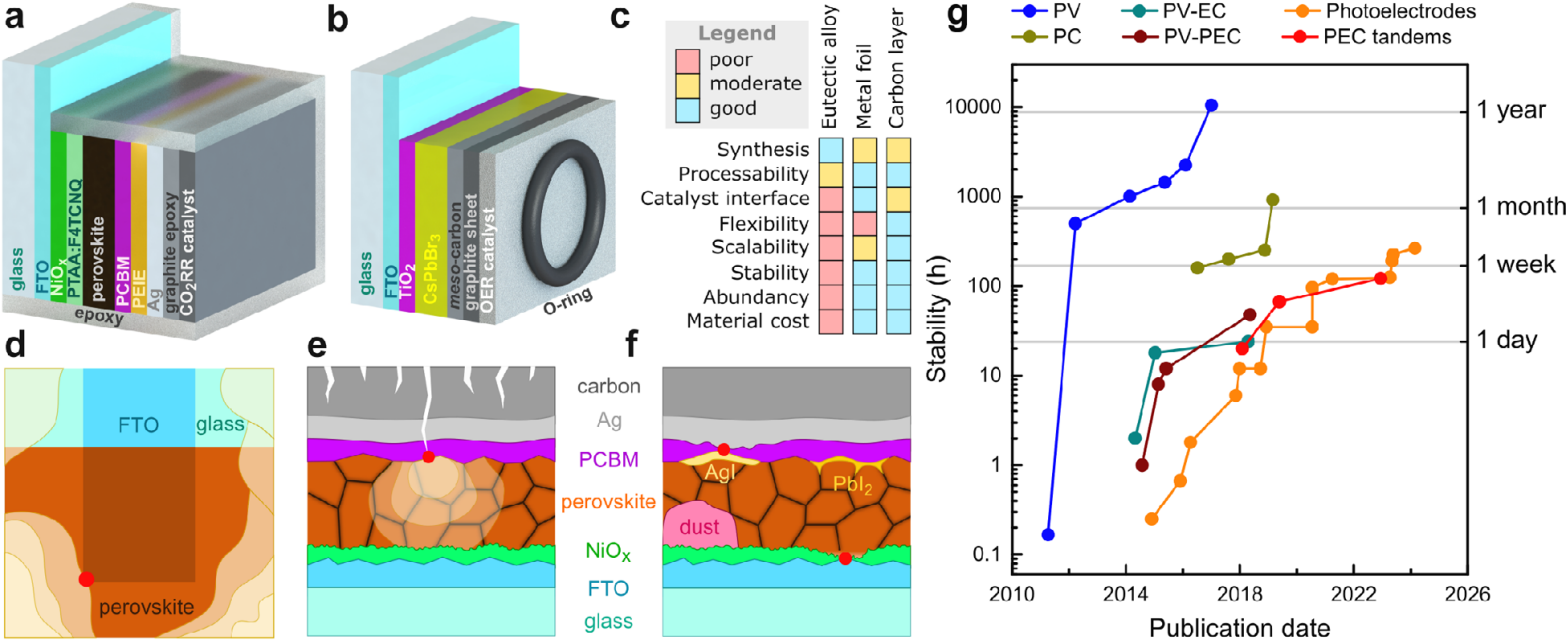
Moisture encapsulation of perovskite photoelectrodes and degradation mechanisms. (a,b) Examples of typical electrode architectures: a ‐ epoxy‐encapsulated photocathode, b ‐ photoanode with an O‐ring. (c) Comparison between conductive moisture encapsulants. (d–f) Degradation mechanisms: d ‐ gradual lateral moisture infiltration, e ‐ cross‐plane moisture infiltration through device pinholes, f ‐ internal device degradation due to film defects, phase segregation, and halide reaction with the metal contacts. Red dots indicate the origin of abrupt photocurrent decay. (g) Reported stabilities of lead halide perovskite light harvesting systems (PV‐PEC = perovskite PV cell wired to a submerged photoelectrode; PC = photocatalyst nanoparticle). A similar trend is observed between technologies, signaling that buried‐junction perovskite photoelectrodes may soon reach the year‐long operation of PV devices.

As hinted earlier, charge separation within a perovskite photoelectrode occurs between the electric contacts of the buried PV device, for instance a transparent FTO or ITO layer and a metal electrode. As perovskites are intrinsic semiconductors, the charge flow will be directed depending on the device architecture [1]. An inverse‐structure (also called p‐i‐n) perovskite device of an FTO|HTL|perovskite|ETL|metal stacking will direct holes toward the catalyst surface, forming a photocathode for proton or CO_2_ reduction (Figure 2a) [14]. On the other hand, a regular‐structure (n‐i‐p) device following an FTO|ETL|perovskite|HTL|metal stacking will direct holes toward the catalyst surface, forming a photoanode for O_2_ evolution [18] or organic oxidations [19] (Figure 2b).

One key advantage of perovskite devices is their high photovoltage reaching above 1 V. This translates to early photoelectrode onset potentials, which can compensate for high overpotentials of electrochemical reactions like CO_2_ reduction or water oxidation. Qualitatively, the photovoltage extracted across the perovskite device depends on the energy difference between the valence band (VB) level of the HTL and the conduction band (CB) level of the ETL. Accordingly, a high photovoltage is obtained when there is close band alignment between the energy levels of the charge transport layers and those of perovskites (Figure 1c). More rigorously, the extracted photovoltage also depends on band bending and quasi‐Fermi level splitting (QFLS) within the device. While band bending has been discussed at length for classical semiconductor‐electrolyte interfaces [8, 20], more factors come into play for buried‐junction perovskite photoelectrodes, including the applied potential, electrocatalytic overpotentials, resistive losses, light intensity, perovskite composition, or choice of charge transport layers, which result in a wide variety of band structures [21, 22, 23].

In practice, devices can employ a multitude of interlayers which can act as HTL (e.g., PEDOT:PSS, PTAA, NiO_x_, spiro‐MeOTAD), ETL (TiO_2_, SnO_2_, C_60_, PCBM) [1], or interfacial buffer layers (poly‐TPD [24], PEIE [25], bathocuproine [26]), as explored extensively in the perovskite PV community. For instance, an intermediate PTAA hole transport layer can align closer with the valence band level of a CsFAMA (FA = formamidinium, MA = methylammonium) triple cation mixed‐halide perovskite, yielding a photovoltage reaching 1.1 V, which amounts to an onset potential above 1 V against the reversible hydrogen electrode (RHE) for H_2_ evolution of the corresponding PVK|GE|Pt photocathode (PVK = perovskite PV device, GE = graphite epoxy paste) [27]. The same improvement was obtained when applying this PTAA interlayer on both NiO_x_ and PEDOT:PSS hole selective layers (Figure 2a) [14, 27], whereas, due to its hydrophobicity, a double HTL structure also contributed to a much slower device degradation [27]. A PEIE layer was also reported to improve the work function alignment between organic semiconductors (i.e., PCBM ETL) and the Ag contact [28]. In practice, PEIE acted as a spacer which prevented silver from reacting with iodide ions through defects in the PCBM layer (Figure 2a,f) [25, 29].

As pointed out earlier, device simplicity can improve the applicability of PEC technologies. Hence, one needs to strike a delicate balance between integration of new functional layers and overall device processability. In practice, that means one should reduce the total number of layers and avoid complex (vacuum) deposition chambers, or high‐temperature annealing techniques where possible. For instance, CsPbBr_3_ photoanodes can minimize the number of layers by utilizing a mesoscopic carbon coating as both HTL and conductive contact to the catalyst support (Figure 2b) [18].

### 2.3 Moisture Encapsulation and Degradation Mechanisms

#### 2.3.1 Extrinsic Moisture Infiltration

Degradation of perovskite photoelectrodes occurs primarily through moisture infiltration. Water can reach the perovskite phase either through its top contact layers (cross‐plane, Figure 2e), or it can gradually infiltrate a photocathode laterally (in‐plane, Figure 2d), which decomposes the APbX_3_ (X = halide) structure into AX and PbX_2_ [1]. To overcome these degradation mechanisms, a dual encapsulation approach is commonly employed. An electrically conductive encapsulant is necessary to transfer charges from metal contact to catalyst, whereas a second, inert encapsulant can insulate the device edges and wiring against moisture ingress (Figure 2a).

Care must be taken when matching the properties of individual encapsulants (hydrophobicity, porosity, adhesion), as their interactions with the perovskite device, electrolyte solution, and each other can determine the robustness of the encapsulation. Initial reports of lead halide perovskite photoanodes utilized no protective moisture encapsulation, with a Ni oxidation catalyst deposited directly onto the top Au contact. As the Au|Ni film was in direct contact with the electrolyte solution, infiltration through device pinholes led to a gradual photocurrent decay within 15–30 min [30, 31]. As a result, eutectic alloys [32], carbon coatings [18, 27, 33] and metal foils [34, 35] were introduced as µm‐ to mm‐scale physical barriers, which would prevent cross‐plane moisture infiltration while allowing charge transport (see comparison in Figure 2c). Combinations of these approaches could gradually push the stability of photoelectrodes from minutes to hundreds of hours, as illustrated in Figure 2g.

Early InBiSn eutectic alloy (Field's metal, FM) encapsulants would ideally spread across the PV device surface when heated above their 62°C melting point, before resolidifying during cooldown [32]. In practice, this approach encountered substantial challenges including thermal strain on the device layers, Ag delamination by the FM droplet, or poor adhesion to other metal layers, meaning that only a small percentage of encapsulated devices were functioning. The ratio of working devices (i.e., those lasting standardized 4–10 h chronoamperometry tests) could be increased to about two thirds by introducing ≈1 mm‐thick, pre‐cut FM foils and thermoelectric modules for rapid heating and cooling (30 s forward and reverse current) [25], as well as soldering resin for further adhesion to Ti foils [34]. Yet, FM remained a corrodible metal forming a less ideal interface with the inert epoxy encapsulant. That meant photocathodes would typically only last <10 h [25, 34], while FM oxidation at positive potentials [32] was limiting photoanode applications. Moreover, FM contained indium, a rare element, which would make this technology less suitable for upscaling.

More earth‐abundant and cost‐effective carbon encapsulations were developed in parallel by multiple groups [18, 27, 33]. A first report of a carbon film|Ag paint|carbon film protection allowed an AVA_x_MA_1‐x_PbI_3_ (AVA = HOOC(CH_2_)_4_NH_3_^+^) photoanode to maintain 70% of its initial photocurrent after 12 h [33], whereas an adhesive graphite sheet enabled CsPbBr_3_ photoanodes to produce O_2_ for 30 h [18]. Similar to the metal foils, the compact sheet would fully seal the device surface, allowing only side‐on moisture infiltration [18]. A graphite epoxy (GE) paste was also simultaneously under development following initial studies of conductive carbon‐polymer composites [36]. By mixing the graphite powder and epoxy resin in an appropriate ratio, one could retain the high conductivity of graphite powder above the percolation threshold, while avoiding a brittle composition at high carbon loadings [27]. This paste provided some unique advantages, being spreadable and intrinsically adhesive, which improved its interface to both device surface and catalyst support. Due to its hydrophobicity and close contact to the inert epoxy, the GE paste could also prevent moisture infiltration, with resulting perovskite photocathodes operating for 96 h without signs of moisture degradation [27]. While lateral degradation is sometimes observed, this is primarily due to imperfections of the inert epoxy coating around sharp glass edges and corners (Figure 2d) [14, 34, 37].

**FIGURE 3:**
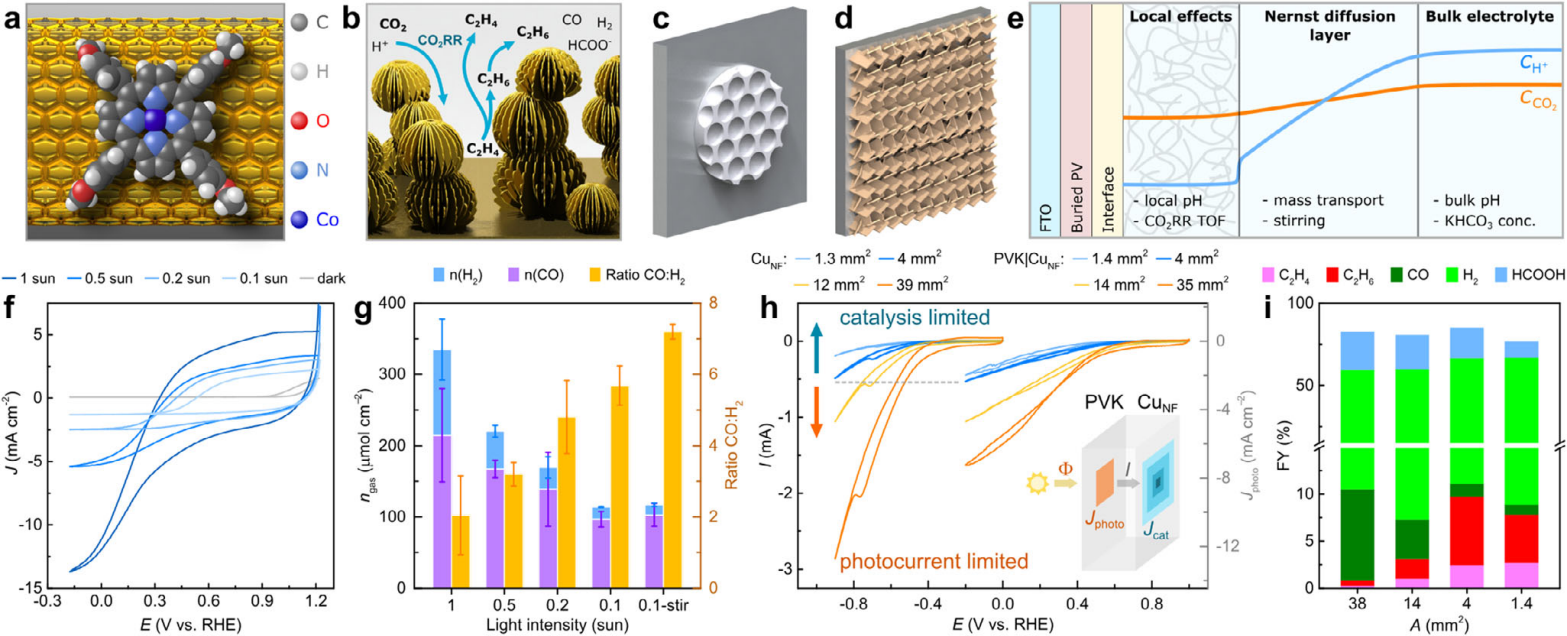
Steering product selectivity in buried‐junction perovskite photoelectrodes. (a–d) Examples of typical electrocatalyst supports: a ‐ Co porphyrin on CNTs for CO_2_RR to syngas, b ‐ Copper nanoflowers (Cu_NF_) on Cu foil for C_2_ hydrocarbons, c ‐ IO‐TiO_2_ grown on Ti foil as scaffold for enzyme immobilization, d ‐ NiFe LDH catalyst on Ni foil for OER. (e) Mass transport and local pH influence on CO_2_RR at porous photocathodes. Bullet points describe factors steering selectivity under each regime, namely inside the pores, near the electrode surface, or in the bulk solution. (f,g) Influence of light intensity on CO:H_2_ selectivity. (h,i) Influence of geometric catalyst area on C_2_ faradaic yield (FY). Both f,g, and h,i showcase the influence of local current density on product selectivity. f ‐ plotted based on data from Ref. [68]. g ‐ adapted with permission from Ref. [14]. b,h,i ‐ reproduced with permission from Ref. [41].

Despite its multiple advantages, one main drawback of graphite coatings is the catalyst interface. While GE paste can readily adhere to catalyst supports (e.g., metal foils, carbon nanotubes), its smooth surface lacking defined binding sites is not intrinsically suited for catalyst loading. For instance, nano‐cracks in the GE surface have been observed over tens to hundreds of hours of operation, which may affect charge transport to 5 nm thin magnetron‐sputtered Pt films [27, 38]. The carbon surface also provided a poor interface to a conventional RuO_x_ H_2_ evolution catalyst leading to a high overpotential and low current density [38]. Since the carbon composite is only stable below 150°C, it cannot act as a substrate for the growth of meso‐ or macro‐porous oxide scaffolds, which require high‐temperature annealing at 200°C–600°C [34, 39], or for hydrothermal growth [40]. Accordingly, such porous catalyst supports or nanostructured catalysts have been conventionally grown onto metal foils (e.g., Ti, Ni, Cu) and subsequently attached to carbon or eutectic alloy layers (Figure 3c,d) [34, 39, 40].

Although embedded metal foils with no intrinsic adhesion may be seen as “very short wires”, they provide the most robust barrier to cross‐plane water penetration, while maintaining the geometry and functionality of classical photoelectrodes (Figure 2c). Here, catalysis occurs over a geometric area (partly) overlapping with the photoactive area of the light absorber, that is, the semiconductor and electrocatalyst do not require separate footprints within an integrated device structure. This unique ability of integrated photoelectrodes to tweak the semiconductor‐to‐catalyst geometric area has been used for instance to improve catalyst selectivity for the CO_2_ reduction reaction (CO_2_RR) [14, 41], as described more in the following section. In recent years, this definition of a photoelectrode has been expanded further to propose device structures with embedded wires fully decoupling light absorption from catalysis (e.g., using regular structure, n‐i‐p PV devices as photocathodes) [42, 43, 44, 45], or the utilization of O‐rings removing the possibility of lateral moisture infiltration altogether (Figure 2b) [45, 46, 47]. While one may argue that these designs stray even further from the functionality of a classical, solution‐immersed electrode, they have resulted in further increases in stability above 200 h [42, 46], as the perovskite PV device is effectively fully blocked from moisture infiltration (Figure 2g). Despite concerns that a bespoke PEC reactor will increase the balance‐of‐cost over a fully‐encapsulated immersed photoelectrode, the practice of utilizing O‐rings to carefully define the active area of sensitive photoelectrodes is also prevalent in the PEC community [41].

A general feature of perovskite photocathodes is that performance is typically compromised once the spacing between the top and bottom conductive electrodes is eroded, i.e., once the device becomes short‐circuited. This corresponds to an abrupt decay in photocurrent, which is sometimes referred to as “catastrophic device degradation”. To this end, carefully designed perovskite photoelectrodes can sometimes display a remarkable film defect tolerance. For instance, 6–16 cm^2^ perovskite PV devices and photoelectrodes sustained currents even in the presence of visible pinholes (unavoidable during spin coating on a larger area), or after the lateral degradation front reached the photoactive area (red dot in Figure 2d) [14, 48]. Such ‘zombie devices’ were again made possible by the hydrophobic PTAA layer, which managed to sustain the device spacing, preventing a complete short circuit.

Although larger device batches can yield statistics on average stability and degradation patterns, it is challenging to predict the onset of “catastrophic” photocurrent decay or the direction of the visible moisture degradation front for individual samples (Figure 2d). This is due to sample heterogeneity, which depends greatly on the quality of manual encapsulation. Nevertheless, optical tracking of the decomposed area, coupled with precise measurements of the device cross‐section, could yield a kinetic rate model of moisture infiltration, which may predict the remaining time until substantial photocurrent decay for a given photoelectrode.

Ultimately, device degradation may raise concerns on the risks of long‐term lead leakage into the environment. To address these, we take again inspiration from the PV community, which developed several complementary strategies to mitigate lead toxicity. These include the introduction of better polymeric encapsulants [49], adding lead‐capturing compounds with electron‐donating functional groups [50, 51], and replacing the photoactive layer with lead‐free compounds such as MASnI_3‐x_Br_x_ [52] or halide double perovskites (e.g., Cs_2_AgBiBr_6_ [53]). Another intrinsic advantage of PEC systems over solar cells is that reactions occur in sealed reactors, which are necessary for product accumulation and collection. These provide an additional physical barrier, as any degradation products are contained within a small water volume which facilitates their recovery and recycling [49, 50, 51].

#### 2.3.2 Intrinsic Photovoltaic Decay

Beyond moisture infiltration, degradation on longer timescales occurs gradually due to imperfections in the film structure. These may involve the presence of film defects, pinholes or clusters, which manifest through an increase in the initial dark currents and lower PV shunt resistance. These are often caused by the surface roughness of the underlying substrate, or by dust grains depositing on the film surface during layer deposition (Figure 2f). Wet deposition techniques like spin‐coating are particularly sensitive to the latter, as microscopic grains can visibly affect nearby solution distribution before annealing. An additional decay in photovoltage and photocurrent can occur over a tens‐of‐hours timescale due to phase segregation, ion migration, photodegradation, and interfacial reactions between the halide ions and reactive metal contacts (Ag, Cu, Al) [29, 54, 55]. This can lead to the formation of insoluble metal halide precipitates (AgX, CuX_2_), phase‐segregated PbX_2_ affecting charge transport at grain boundaries, or composition changes across the perovskite film (Figure 2f) [55, 56].

Most knowledge on inner degradation pathways relies on spectroscopic studies of (incomplete) PV stacks, as perovskite interfaces are buried between a thick glass substrate and an opaque encapsulation layer for perovskite photoelectrodes. A recent study demonstrated that same methods can be deployed for photoelectrocatalysis, by carefully dismantling perovskite photoanodes to expose inner layers. Accordingly, a combination of in situ PL maps, DLCP, ToF‐SIMS, XPS, AFM, KPFM, and cross‐section SEM indicated that charge accumulation due to slow kinetics at the OER catalyst/electrolyte interface may be responsible for higher iodide migration and phase segregation in (FAPbI_3_)_0.95_(MAPbBr_3_)_0.05_ photoanodes, leading to accelerated photocurrent decay compared to an equivalent solar cell [57].

As mentioned earlier, some of these degradation pathways can be mitigated by introducing dual charge selective layers like NiO_x_|PTAA or additional spacers like PEIE [27], which ensure an appropriate coverage of surface defects, or prevent halide ions from reacting with metal contacts. In terms of photodegradation, this usually manifests through a color change across the perovskite film from dark to lighter [37], which does not necessarily translate to a decrease in photocurrent or photovoltage. In recent years, further efforts have been made to tackle this gradual performance delay, by exploring interfacial stabilizers including 2D chiral perovskites [42, 58], or cyclohexylammonium iodide [59].

These approaches drew again inspiration from progress in the PV community, where stabilities of 1000 h are commonly reported under accelerated degradation protocols (e.g., 85°C temperature, 85% humidity) [54], which translate to estimated real‐world operations >5 years [60, 61]. PV cells could even operate beyond 1 year, by using slot‐die coating for conformal film deposition [62], or introducing 2D/3D perovskite interfaces in robust carbon/oxide mesoscopic scaffolds [63]. Alternatively, single crystal perovskite devices can avoid the surface roughness of polycrystalline samples, which promotes phase segregation, favors pinholes, and charge transport layer degradation (Figure 2f). Among many PV studies exploring ways to improve crystallinity [23, 64, 65], a reduction of cross‐plane grain boundaries appears sufficient to achieve effective charge extraction, which bypasses the strict limitations of growing a device‐wide single crystal [66]. While these interfacial design strategies show great promise, with stabilities now reaching 265 h [42], one must keep in mind that photoelectrodes are tested under aqueous solutions, where a much higher local concentration and ionicity of water can accelerate moisture permeation and corrosion. Therefore, effective encapsulation remains the dominant factor for matching PV cells in long‐term operation (Figure 2g).

### 2.4 Perovskite‐Inspired Materials and Device Architectures

Photoelectrodes based on moisture‐sensitive metal halide perovskites present an ideal model system to gain insights into device encapsulation, catalyst integration, and overall reactor engineering. These findings can be easily transferable to a broad range of promising light absorbers, which may have similar optoelectronic properties yet a better intrinsic stability. For instance, H_2_ evolution could be achieved for 500 h by applying the graphite paste encapsulant to a NiO_x_|BiOI|ZnO oxide‐based device structure [38], whereas a similar stability of up to 300 h was recorded for a HER photocathode employing a PCE10:EH‐IDTBR organic photovoltaic (OPV) blend [67].

## 3 Catalysis

### 3.1 Reaction Scope

Another key advantage of buried‐PV photoelectrodes is their versatility, as light absorbers can be interfaced to a wide range of catalysts and catalyst supports through the conductive encapsulation methods introduced above. For instance, perovskite devices have been interfaced to Pt films for hydrogen evolution reaction (HER) [25, 27], to molecular cobalt porphyrin [68] and phthalocyanine [69] catalysts immobilized on carbon nanotubes (CNTs; Figure 3a) for the CO_2_ reduction reaction (CO_2_RR), to enzymes immobilized on hierarchically nanostructured inverse‐opal (IO) TiO_2_ scaffolds for HER [34] and CO_2_RR (Figure 3c) [70], to Ru‐loaded titanate nanosheets for nitrate reduction to ammonia [71], or to metal (alloy) catalysts for CO_2_RR to C_1_ (CO [26], formic acid [24]) and C_2+_ products (ethanol, *n*‐propanol [39], ethylene, ethane [41]; Figure 3b). On the oxidative side, photoanodes have employed molecular iridium binuclear catalysts bound to graphite sheets [18], NiFe layered double hydroxide (LDH) catalyst grown onto NiFe alloy (Figure 3d) [37], or NiFeOOH deposited onto graphite sheet [47] and Ni foil substrates [46, 72] for the oxygen evolution reaction (OER), as well as CoNiFe LDH grown onto Ni foils for the conversion of organic substrates like glucose, glycerol, ethylene glycol into value‐added chemicals [19].

### 3.2 Mass Transport and Local pH Effects

One property of classical photoelectrodes maintained by buried‐junction perovskite electrodes is their geometry. As mentioned earlier, light harvesting and catalysis occur on opposite sides of the integrated photoelectrode panel, hence, one side of the electrode is blocked by the transparent glass substrate, becoming inaccessible for substrate diffusion (e.g., protons, or CO_2_). That means, mass transport to and from the catalyst surface can vary substantially in integrated photoelectrodes, compared to freestanding porous catalysts supports. This has been shown to induce substantial changes in CO_2_RR selectivity between freestanding dark electrodes and their corresponding photocathodes for CNT‐immobilized cobalt porphyrin [68] and carbon paper immobilized Cu nanoparticles [41].

These changes in mass transport regime can also be used to one's advantage when designing buried‐junction photoelectrodes. As explored in the electrocatalysis literature, an increased catalyst and substrate porosity can improve CO_2_RR selectivity by inducing a higher local pH within the pores. A depletion in the local proton concentration reduces the competing HER, whereas the concentration of solvated CO_2_ is maintained steady through the CO_2 (aq.)_ + H_2_O ⇄ H_2_CO_3_ ⇄ KHCO_3_ equilibrium (Figure 3e) [73, 74]. Looking beyond simple C_1_ products, a more porous catalyst may also induce changes in the CO_2_RR selectivity of C_2_ hydrocarbons, as ethylene can for instance readsorb on the catalyst surface for further reduction to ethane (Figure 3b) [75, 76].

### 3.3 Photocurrent Density

The current flow though the photoelectrode can also substantially influence CO_2_RR selectivity. In case of electrocatalysis, the current is primarily controlled by the applied potential. Hence, its absolute magnitude is limited by a combination of factors including mass transport, catalyst loading (i.e., the number or active sites), or its intrinsic turnover frequency (TOF). In case of photoelectrodes, the current is additionally limited by the photocurrent supplied by the light absorber, which in turn depends on the incoming photon flux and external quantum efficiency of the device (i.e., photon‐to‐charge efficiency across the solar spectrum) [41]. That means, the photocurrent of a buried‐PV electrode is simultaneously set through both applied potential and light intensity (Figure 3f).

Due to its modular structure, such mechanistic insights can be directly translated to a perovskite‐embedded photocathode. For instance, an increase in the CO:H_2_ selectivity was observed when decreasing the current density of a perovskite photocathode with a CNT‐immobilized cobalt(II) *meso*‐tetrakis(4‐methoxyphenyl)porphyrin (abbreviated PVK|CoMTPP@CNT) [68]. A comparable amount of CO was produced over 4 h at 0 V versus RHE likely due to mass transport limitations, whereas the amounts of H_2_ decreased substantially from 1 sun (100 mW cm^−2^, AM1.5G spectrum) to 0.1 sun irradiation. In this case, a low catalyst loading ensured that most active sides were found within the porous CNT sheet, whereas its CO_2_RR TOF meant that a higher photocurrent density could only contribute to proton reduction [68].

This interplay between mass transport, local equilibria, current density and product selectivity was taken into account when redesigning a lightweight PVK|CoMTPP@CNT electrode [14]. Following earlier findings, the geometric area of the CoMTPP@CNT catalyst was increased threefold, achieving a better coverage of the device footprint area. This enabled a perovskite photocurrent dilution over a larger catalyst area, provided more active sites and reduced CO_2_ depletion nearby the electrode surface. This resulted in a CO:H_2_ ratio of 2:1 under 1 sun irradiation (a 9× improvement), as well as a CO:H_2_ ratio of 7.2:1 at 0.1 sun under solution stirring (Figure 3g) [14], matching the best selectivity obtained for the freestanding CoMTPP@CNT catalyst under stirring [68].

While perovskite photocathodes can sustain high C_1_ product selectivities under varying catalyst geometries [68], a precise current matching becomes more crucial for the sensitive C_2_ hydrocarbon synthesis. Accordingly, a recent study demonstrated that an optimal ethane + ethylene faradaic yield of 10% could be attained when the photocurrent passing through the catalytic area of the photocathode matched the dark current from electrocatalysis (Figure 3h,i) [41]. Since light harvesting is spatially decoupled from catalysis, that means both serially connected components experience different current densities for the same absolute photocurrent. If the catalyst area is larger, this will dilute the photocurrent from the perovskite PV device, resulting in a lower catalytic current density. If the catalyst area is smaller, the light absorber will again induce an excess current density through the catalyst, leading to H_2_ evolution (Figure 3h) [41].

## 4 Unassisted PEC Devices

### 4.1 Artificial Photosynthesis

**FIGURE 4:**
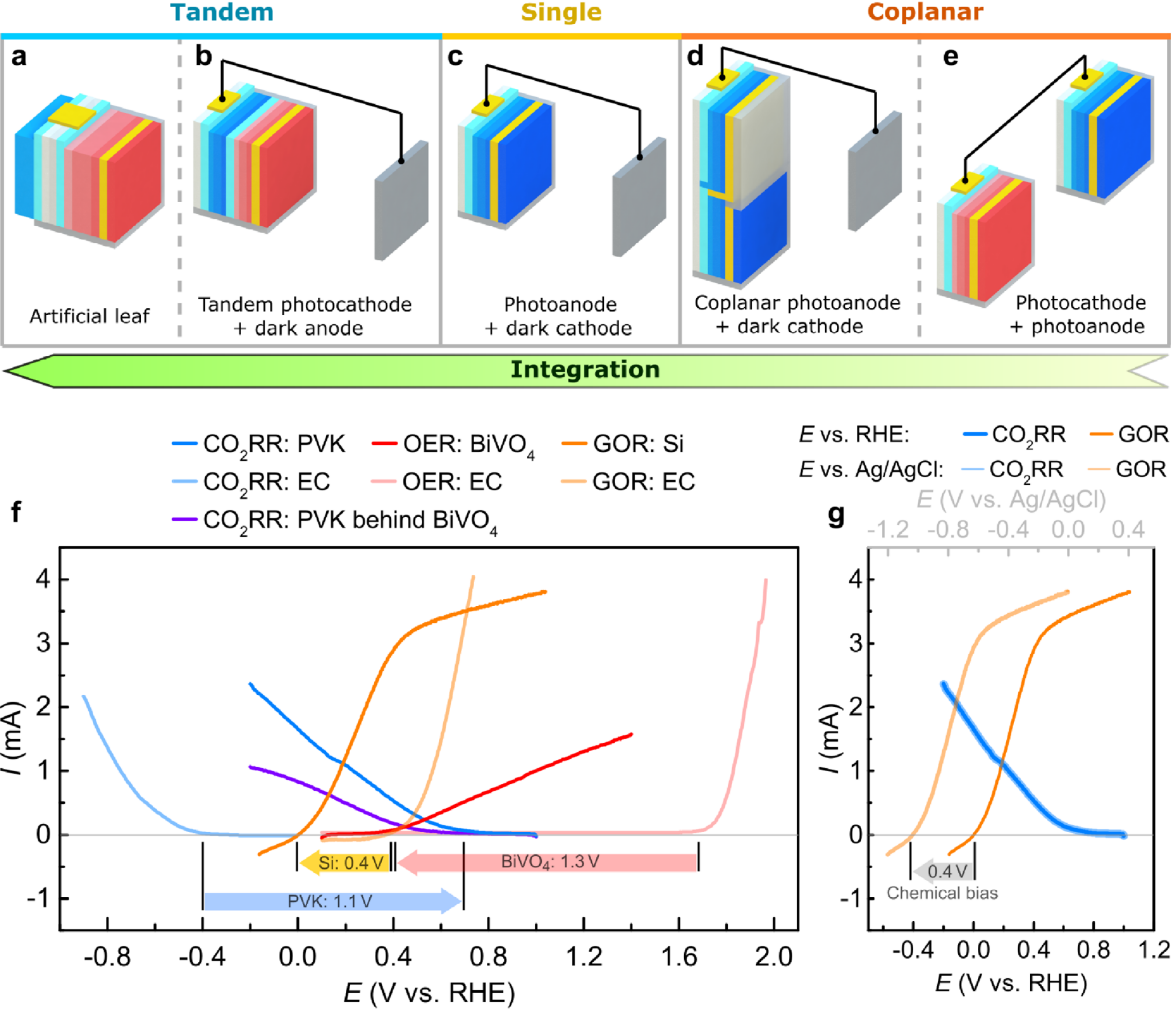
Designs and performance of unassisted perovskite‐based PEC devices. (a–e) Device types based on design and degree of integration: a ‐ artificial leaf, b ‐ tandem PV photoelectrode wired to dark counter electrode, c ‐ single light absorber coupling reductive reactions (HER, CO_2_RR) to organic oxidations, d ‐ coplanar photoanode coupled to a dark cathode, e ‐ side‐by‐side device combining a photoanode with a photocathode. (f) Overlap between the linear sweep voltammograms of perovskite photoelectrodes with those of common inorganic photoanodes and dark electrodes. (g) Effect of chemical bias on the overlap of LSV traces, i.e., on the bias‐free operating photocurrent.

Compared to traditional silicon, Cu_2_O or organic‐based photoelectrodes, metal halide perovskite devices can reach high photovoltages above 1 V [68]. This translates in photoelectrodes with early onset potentials (Figure 4f), which makes them ideal for unassisted solar fuel synthesis beyond water splitting. For instance, perovskite‐based photocathodes display early onset potentials of around 1 V versus RHE for HER and 0.7 V versus RHE for CO_2_RR. This results in good overlaps with the photocurrent traces of oxide photoanodes for OER (e.g., BiVO_4_, WO_3_, Fe_2_O_3_) [77], resulting in bias‐free tandem PEC devices (Figure 4f). These devices, which store solar energy in chemical bonds by converting water and CO_2_ into energy vectors like H_2_, CO, and formic acid, are often referred to as artificial photosynthesis systems.

Based on the reaction scope and choice of electrodes, unassisted PEC devices can be assembled in different configurations. Most commonly, perovskite photocathodes have been coupled to BiVO_4_ photoanodes in a back‐to‐back tandem PEC configuration. These devices can be wired to an external potentiostat in a 2‐electrode configuration to trace photocurrent stability and faradaic yield over time. Alternatively, the two electrodes are directly connected through an embedded Cu stripe, resulting in a so‐called monolithic, wireless, or standalone “artificial leaf” (Figure 4a) [25]. This photosynthesis‐inspired design can better harvest the solar spectrum by using complementary light absorbers. The wide bandgap oxide photoanode utilizes blue light to oxidize water, whereas the remaining red light filters through to the perovskite photocathode to perform reductive reactions. This arrangement minimizes the photoactive footprint area of the device, resulting in a higher theoretical efficiency over equivalent side‐by‐side devices. Accordingly, perovskite photoelectrodes employing state‐of‐the‐art tandem PV devices can reach unassisted STH >15% [78] or even >20% [79] when wired to an appropriate counter electrode (Figure 4b).

In practice, a side‐by‐side, coplanar PEC device configuration is necessary for opaque photoelectrodes (Si [41], Cu_2_ZnSnS_4_, buried‐junction perovskite [19]; Figure 4e) or electrode substrates (Ti‐deposited BiVO_4_) [14]. Light scattering through nanostructured oxide photoanodes can further decrease the light flux reaching the perovskite photocathode, reducing the photocurrent overlap and overall device STF (Figure 4f) [25]. Accordingly, multiple reports have demonstrated high solar‐to‐hydrogen conversion efficiencies (STH) >10% by coupling perovskite photocathodes side‐by‐side with perovskite photoanodes (Figure 4e) [42, 79, 80, 81]. A similar STH efficiency can be achieved with coplanar dual‐perovskite photoelectrodes, where two PV devices are serially connected and encapsulated on the same conductive substrate (i.e., a single physical panel; Figure 4d) [44, 72]. Conceptually, these designs represent more integrated alternatives to the original 2014 PV‐EC prototype, where two perovskite solar cells wired to NiFe LDH electrocatalysts could demonstrate unassisted water splitting at 12.3% STH [5].

### 4.2 Photoelectrocatalytic Organic Synthesis

One emerging alternative is replacing the thermodynamically challenging OER with organic oxidations like the glycerol oxidation reaction (GOR). The latter occur at electrochemical potentials as low as 0.1–0.3 V versus RHE [41], which can avoid the need for dual light absorber systems. Hence, single perovskite photocathodes have been shown to supply enough photovoltage to couple HER and nitrate reduction to glycerol [71], glucose, ethylene glycol [82], or lignocellulosic biomass oxidation [83] at high product rates (Figure 4c). These organic substrates showcase the potential of converting industrial byproducts and household waste into value‐added chemicals, while simultaneously producing pure H_2_ at high rates of >10 mA cm^−2^. Products like gluconic acid [19], glycolic acid [82], glycerate, lactate, acetate, formate [41], vanillin and acetovanillone [83] are valuable for the food, cosmetic, and pharmaceutical industries. In the future, this approach may expand to the selective conversion of complex molecules for the fine chemical industry (e.g., target specific functional groups), providing an ideal market niche for this technology.

While HER is favored by the strongly alkaline conditions required for electrochemical organic oxidations, a neutral‐pH electrolyte is needed to maintain CO_2_ solvation and prevent CO_2_ capture as carbonate for CO_2_RR systems [41]. This means that 2‐compartment PEC cells need to employ a bipolar membrane separating the neutral‐pH cathodic from the alkaline anodic sides. One factor that should be kept in mind here is the additional chemical bias occurring between the two compartments [84]. This has an overall favorable effect on the reaction rate, as indicated by the improved current trace overlap obtained when plotting both (photo)electrodes on the same Ag/AgCl scale (Figure 4g). Note that the potential of the reference Ag/AgCl/KCl_(sat.)_ electrode does not vary as a function of pH, hence the pH correction applied when converting to the RHE scale does not apply.

Despite a facile oxidation reaction and chemical bias, a dual‐light absorber system may be required if the targeted CO_2_RR reaction requires high overpotentials to attain sufficient product selectivity. For instance, perovskite photocathodes were coupled to Si nanowire photoanodes for overall C_2_ hydrocarbon synthesis and GOR [41]. In this case, electrocatalytic GOR with a PtAu catalyst has an onset potential around 0.4 V versus RHE, which already provides a 1.3 V gain over the 1.7 V onset potential of an amorphous TiCoO_x_ catalyst for OER. A Si nanowire photoanode could provide an additional 0.4 V photovoltage, whereas its high current compared to the wide bandgap BiVO_4_ meant that the overlap between the cathodic and anodic photocurrent traces was greatly improved (Figure 4f). More importantly, this improved overlap occurred at an early crossover point of 0.1–0.2 V versus RHE, meaning that the unassisted PEC device could sustain a C_2_ hydrocarbon selectivity close to that observed for individual perovskite photocathodes in a 3‐electrode configuration [41].

Alternatively, the same photovoltage may be supplied by wide bandgap perovskite devices with high bromide contents (e.g., CsPbBr_3_, FAPbBr_3_), which display open circuit voltages above 1.3 V. For instance, FAPbBr_3_ photoanodes have demonstrated an early average onset potential of −0.066 V versus RHE, a photocurrent density of 8.7 mA cm^−2^ at 1.23 V versus RHE and stability beyond 120 h for oxygen evolution [37]. While this photoanode provides just enough driving force for overall water splitting, its photovoltage would be sufficient to couple CO_2_‐to‐multicarbon conversion to organic oxidations. In this case, either photoanodes could be directly interfaced to organic oxidation catalysts (e.g., PtAu alloy for glycerol oxidation [41]), or new CO_2_RR photocathodes could be designed to utilise the wider bandgap semiconductor. Both designs can be coupled to a second photoelectrode for additional driving force. However, special care must be taken with the spectral coverage of both light absorbers in a tandem “artificial leaf” configuration, as Br‐rich perovskite compositions with bandgaps around 2.2–2.4 eV absorb at wavelengths <550 nm [37], which overlap with the absorption ranges of common metal oxide photoelectrodes (e.g., BiVO_4_: 2.5 eV, λ < 500 nm [85]).

## 5 Reactor Engineering and Scaleup

### 5.1 Addressing Scalability Challenges

Despite higher STF efficiencies, (perovskite) PV‐electrolysers require additional wiring, electronics, or bulky (flow) reactors, which increase system complexity, and therefore operating costs [86]. PEC systems have the unique potential to balance the performance of PV‐electrolysis with the simplicity of photocatalytic powders [77], which makes them highly promising for solar fuel applications. Although intricate PEC prototypes can demonstrate STH efficiencies >10% (widely considered a competitive target for commercialisation), until recently, only few studies explored their scalability beyond a cm^2^ laboratory scale, which raised concerns regarding applicability compared to more established PV technologies. Here, we will make an argument that buried‐junction perovskite photoelectrodes are in fact ideally suited for real world applications, due to their excellent thin‐film processability and compatibility with modern deposition techniques.

To successfully scale up PEC systems, one must first understand their limitations. From their inception, monolithic artificial leaves have been notoriously known for displaying lower STF values compared to their separated 2‐electrode counterparts. A main reason for this efficiency loss is a pH difference build‐up between both sides of the device, which contributes to an additional electrochemical overpotential [87]. This pH difference has been shown to be most prominent in stagnant neutral pH solutions, whereas strongly acidic or basic solutions [88], or electrolyte flow [89, 90] have been shown to mitigate these losses. One proposed alternative was introducing perforated large scale‐electrodes, which would let charges equilibrate across the physical electrode barrier [91]. Similarly, modular arrays have been proposed where either gaps, or ion selective membranes (e.g., Nafion, Selemion) are located between artificial leaves to allow pH equilibration [14, 26].

**FIGURE 5:**
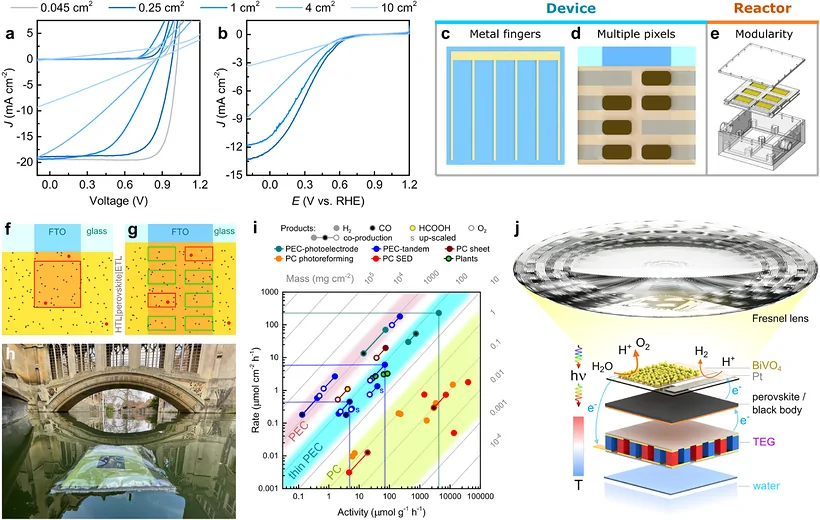
Challenges and strategies in upscaling perovskite‐based PEC systems. (a) Effect of series and shunt resistance on the current‐voltage traces of perovskite PV cells of increasing photoactive areas. (b) LSV traces of the equivalent perovskite photocathodes. (c–e) Strategies to avoid resistive losses: c ‐ metal fingers, d ‐ multiple pixels operating in parallel, e ‐ modular reactors housing arrays of smaller PEC devices. (f,g) Effect of randomly distributed defects on the viability of: f ‐ single‐pixel, g ‐ multiple‐pixel devices. (h) Floating perovskite‐BiVO_4_ artificial leaf. (i) Comparison between the product rate and activity per gram of solar fuel technologies. (j) Integration of PEC reactors with thermoelectric generators for waste heat harvesting under concentrated sunlight. a,b ‐ adapted with permission from Ref. [25]. d,f,g ‐ adapted with permission from Ref. [38]. e ‐ reproduced with permission from Ref. [26]. i ‐ adapted with permission from Ref. [14]. j ‐ adapted with permission from Ref. [48].

Another substantial challenge toward manufacturing large scale PEC devices is posed by the sheet resistance of the conductive substrates, which can limit photocurrent output. While resistive losses are less prominent on a small scale, a linear, ohmic behavior of the current‐voltage traces becomes visible at high absolute photocurrents around 100 mA [25]. In case of perovskite PV devices, this sheet resistance translates to a gradual decrease in fill factor with increasing area, which ultimately diminishes the shot‐circuit photocurrent density (J_SC_) (Figure 5a). Although the open circuit voltage (V_OC_) remains constant, this does translate to a similar loss in photocurrent for corresponding perovskite photoelectrodes (Figure 5b) [25]. To address this, practical solutions have been the growth of semiconductors on highly conductive metal substrates (e.g., BiVO_4_ on Ti foils [14] and felts [92]), or the patterning of metal fingers on transparent glass substrates for better charge collection (e.g., electrodeposition of Ni lines [93] or thermal evaporation of Au fingers [94] on FTO; Figure 5c). While these solutions suit simple semiconductor coatings, there have been challenges in translating this strategy to the multilayered perovskite devices. Our initial attempts with Cu fingers led to shorted devices, likely due to film defects caused at the step edge between Cu film and FTO. However, an approach including Ni fingers may prove successful, as the Ni surface can oxidize to NiO_x_, which was already proven as an effective hole selective barrier on rough substrates [25].

Moreover, the sheet resistance can induce a potential drop across the conductive substrate, hindering the homogeneous electrodeposition of semiconductors and electrocatalysts like BiOI (BiVO_4_ precursor) and RuO_x_, respectively. To this end, practical solutions have been the improvement of ohmic contact through better clamping over a larger area, or the design of custom electrodeposition baths which minimize distance between electrodes [14]. For BiOI, the standard Pt mesh counter electrode has also been replaced by a wide‐area graphite foil electrode, in order to maintain a steady pH gradient across the benzoquinone electrodeposition solution [14].

One advantage of perovskite photoelectrodes is that the fill factor of linear sweep voltammograms (LSVs) is mainly governed by the solution resistance (i.e., *iR* drop) and electrochemical overpotential rather than much faster increasing PV currents. That is, one does not require state‐of‐the‐art PV devices with record power conversion efficiencies (PCE) to perform electrochemical reactions at high rates. For those PV devices, PCE is mainly determined by a good FF, rather than substantial increases in J_SC_ and V_OC_. That means, one can design devices with somewhat thicker charge selective layers, which may reduce the J_SC_ and FF, but overall provide a better reliability and stability over a larger area [25].

Besides ohmic losses, shunt resistance can also affect the device performance on a larger area. In this case, pinholes and defects cause a leakage current, which affects the V_OC_, J_SC,_ and FF of perovskite PV devices (see change of V_OC_ and dark currents with area in Figure 5a). While it is challenging to avoid dust‐induced film defects when spin‐coating on larger areas, dual PEDOT:PSS|PTAA hole transport layers and interfacial PEIE layers have proved successful in preventing degradation [14], as detailed earlier in Section 2. Despite its complex setup, one technique that stands out in this case is the vacuum deposition, which allows the simultaneous evaporation of multiple precursor salts (e.g., CsBr, FAI, and PbI_2_) to produce perovskite films of defined thickness and composition, as well as subsequent layer deposition in a vacuum environment. Its reproducibility means that over 100 devices can be reliably deposited, barring the presence of organic impurities in the deposition chamber [26].

Beyond device level, reactor engineering is also crucial for translating solar fuel technologies to real world applications. Critics of these technologies often quote the product collection over a large area and the generation of explosive H_2_ and O_2_ product mixtures as main drawbacks. However, recent studies of 12–100 m^2^ photocatalytic panels have shown that flow systems coupled with gas separation membranes can effectively collect H_2_ and O_2_ products [95, 96], whereas the overall system is robust to accidental combustion [95]. As proposed earlier, a better product separation may be achieved by physically integrating devices within ion selective membranes, or tightly clamping them between separate anodic and cathodic compartments. Another way to improve product separation is by taking advantage of gaseous and liquid phases. For instance, H_2_ produced at the photocathode can be collected from the reactor headspace, while soluble organic oxidation products accumulate in the electrolyte solution.

In practice, one key challenge encountered by upscaled prototypes is reactor leakage [26], which can lead to air infiltration and product loss. An uneven device surface can further induce product crossover between anodic and cathodic compartments, which can lead to back reactions such as O_2_ reduction on a Pt surface. Another challenge is the intermittency of atmospheric conditions including light intensity, temperature, and pressure. Light intensity can vary substantially during the day, reaching 1 sun (i.e., 100 mW cm^−2^ direct irradiation) around noon, but only 0.1–0.2 sun during cloudy overcast weather (i.e., diffuse irradiation) [26, 97]. Sunlight irradiation only reaches around 0.5 sun during winter [97], making tests at different light intensities essential to model the performance of PEC devices under cold climates at higher latitudes. In contrast, high temperatures attained under operation in warm climates have been shown to contribute to an accelerated device degradation or overpressure in the reactor chamber [98], whereas day‐night temperature and pressure changes can also wear the mechanical reactor joints [26]. While scalability studies are still at a very early stage, the increasing number of prototypes including integrated PEC, PV‐EC or PC systems over the past 5 years shows promise that solar fuels technologies may soon approach practical applications [96, 99]. Nevertheless, broader, more concentrated efforts are required to design and potentially even standardize robust solar reactors, which will take an interdisciplinary combination of expertise between chemists, material scientists, and engineers.

### 5.2 Emerging Strategies Toward Upscaling

Besides these strategies, a number of original approaches have also been proposed over the past years to improve the scalability of buried‐junction PEC systems. These take advantage of the modularity, processability, defect tolerance, and potential toward integration of thin‐film perovskite devices.

One key advantage of artificial leaves is their modularity. To cover a wider surface, one does not need a single large device. Instead, this can be replaced by an array of smaller devices of equivalent total area (Figure 5e) [26]. These can avoid resistive losses and pH gradient build‐up as described earlier. Since each panel operates independently, the degradation of one device does not translate to a loss in performance for other devices, which provides a substantial advantage over PV modules. A similar concept can be introduced when designing individual photoelectrodes. As described earlier, a combination of substrate sheet resistance and film defects commonly lead to a decrease in PV device photovoltage and photocurrent with increasing area, which translate to similar effects for the equivalent photoelectrodes. By wiring smaller perovskite photoelectrodes in parallel, one can sustain the high photovoltage of individual electrodes, while the cumulative photocurrent will be a multiple of the active area, hence, the overall array will also sustain the high photocurrent density of individual electrodes [45, 59, 80].

This design can be further integrated on the same electrode surface, resulting in the concept of pixelated photoelectrodes (Figure 5d) [38]. In this case, a preselection is required to weed out the defective “pixels”, or small solar cells, a term directly borrowed from multiple‐pixel perovskite PV devices. For this purpose, we will first differentiate between minor film defects—those contributing to negligibly small current leakage, and major film defects—large, often visible film imperfections that lead to a shorted device (Figure 5f,g). While the statistical distribution of minor and major film defects across a given surface area is the same, it is more likely to encounter a major film defect on a larger area. Therefore, in practice it is more likely to encounter shorted single‐pixel devices when fabricating them on a larger area (Figure 5f). By fabricating an array of smaller devices across the same substrate, one can confine the negative effect of major pinholes to a smaller active area (Figure 5g). For instance, “perovskite‐inspired” BiOI photocathodes could demonstrate almost two‐times higher photocurrents and a 0.2 V earlier onset potential compared to single‐pixel counterparts of same photoactive area (≈0.25 cm^2^). This earlier onset and sustained photocurrent allowed BiOI—BiVO_4_ tandem devices to attain a 240 h stability for unassisted water splitting [38].

Solar harvesting systems also experience efficiency losses from photons with energies below the semiconductor bandgap (e.g., non‐absorbed IR radiation), or from thermalization above the bandgap. Hence, over 50% of photon energy is lost in the form of waste heat, as governed by the Shockley‐Queisser limit [100]. One alternative is taking advantage of these thermal effects to accelerate reaction kinetics [101]. Such systems rely on concentrated sunlight, which is commonly obtained with Fresnel lens, parabolic mirrors [102], or optical waveguides [98]. The use of solar concentration reduces the photoactive area required for the reaction. However, concentrated sunlight also requires complex solar tracking devices with active cooling, as well as direct sunlight irradiation, which may restrict the applicability to specific climates.

Another alternative is integrating thermoelectric modules with PEC systems, in order to harvest this waste heat into an additional electrical bias [103]. For instance, commercial Peltier modules could utilize the temperature difference between a heated PEC reactor and a room temperature water bath (acting as a heat sink) to supply up to 0.5 V under 5 sun concentration (Figure 5j) [48]. This enabled a 1.7× boost in the photocurrent of perovskite‐BiVO_4_ devices at 5 sun irradiation. The additional thermoelectric (or Seebeck) voltage proved even more useful in case of photoelectrodes with a less ideal overlap, resulting in a 3.7× enhancement under 5 sun for perovskite‐Fe_2_O_3_ devices, or 30× enhancement compared to the PEC photocurrent under 1 sun irradiation. More impressively, the TE photovoltage was enough to achieve unassisted water splitting with a single BiVO_4_ photoanode at a light concentration as low as 2 sun. Taking thermal degradation into account, higher light concentrations are in principle possible, up to a temperature below the water boiling point of 100°C. As TE voltage is proportional to the temperature difference, this could lead to an additional bias voltage above 1 V, depending on the exact TE module specifications (pairs of legs, thermal and electrical conductivity) and configuration (single‐ or multi‐stage).

A final example is the emerging concept of “floating artificial leaves”, which is poised to benefit the most from the processability of perovskite PV and PEC devices (Figure 5h) [14]. Conventional PEC prototypes are fabricated on 2–3 mm thick glass substrates and utilize thick epoxy coatings, resulting in small, bulky devices with photoactive area <1 cm^2^. This intrinsic design limitation means that devices remain far from the low weight, abundancy and flexibility of plant leaves, hindering their practical implementation. However, the photoactive and catalytic layers are only nano‐ to micrometer‐thin. Hence, by depositing these onto thin metal and plastic substrates, devices could be obtained which are thinner, lighter, flexible, and scalable. For instance, perovskite devices could be deposited on 127 µm thin ITO‐coated PET sheets (PET = polyethylene terephthalate). A 10 µm thin parylene‐C coating was added to protect the device edges, whereas moisture protection was achieved through a 100–300 µm‐thin graphite epoxy coating. On the anodic side, BiVO_4_ was grown onto 25 µm‐thin Ti foils, whereas H_2_SO_4_ treatment was needed to remove the native TiO_2_ surface layer before BiOI precursor electrodeposition [14]. This concept could be recently translated to layered Cu_2_O—BiVO_4_ devices [104], emphasizing the versatility of this technique for buried‐junction electrodes.

Due to a low weight of 30–100 mg cm^−2^, these devices managed to achieve a performance similar to natural leaves in terms of both product rate (amount normalized by the photoactive area) and activity (product amount normalized by the weight of the device). Moreover, lightweight perovskite photocathodes could retain the high product rate of wired systems, while matching the high activity of photocatalyst powders (Figure 5i). Looking back at the qualitative compromise of solar fuel technologies between high performance, high complexity and cost (PV‐electrolysis) or low performance, low complexity and cost (photocatalyst dispersions) [77], these powerful metrics of product rate and activity provide quantitative proof for the unique potential of lightweight PEC systems. Moreover, these floating PEC devices weigh 15 times less than conventional artificial leaves, or >100× less than state‐of‐the‐art floating PV farms which still require heavy metal frames and bulky floaters to operate [105]. While complex PV‐electrolysis flow systems can reach 30% STH efficiency [106], lightweight artificial leaves can reduce material costs 1000‐fold, decreasing requirements on efficiency or device lifetime. As shown recently in the silicon PV industry, market adoption is driven by cost reduction, rather than marginal efficiency gains [107].

This architecture redesign also led to novel device functionality. Accordingly, due to their low weight, bubbles formed during operation could make devices float on the water surface like artificial “lotus leaves”. This could also be performed outdoors, using sealed plastic bags with a rubber septum as the cheapest, lightest reactor one can envision (Figure 5h). As water covers 70% of Earth's surface, the deployment of floating solar fuel farms may prove key in bringing this technology to the market, finding untapped niches for deployment in remote coastal areas or islands, where their low weight and flexibility makes them ideal for deployment [14].

The processability and defect tolerance of thin‐film PV technologies also makes them ideal for industrial scale fabrication. These features enable a rapid deposition of high efficiency, µm‐thin devices on rigid and thin film substrates, using a wide range of solution and vacuum‐based techniques, which can provide a key advantage over mature technologies such as metalorganic vapour phase epitaxy needed for III‐V semiconductors [13, 108]. While the complexity of layered perovskite devices compared to classical photoelectrodes may raise concerns in the PEC community, modern fabrication techniques have been mastered by perovskite PV groups for sheet‐to‐sheet and roll‐to‐roll device fabrication [109]. Such deposition techniques include slot‐die coating, blade coating, screen printing and spray coating of perovskite devices, which can be nowadays performed in commercially available machines [110, 111]. Going back to the design considerations presented in Section 2, one such setup is ideally only 2–3 steps away from roll‐to‐roll fabrication of scalable perovskite photoelectrodes. The only coatings differentiating a photoelectrode from a solar cell are the (conductive) encapsulant and the catalyst layer.

### 5.3 Outlook on Technological Translation

It is worth emphasizing that perovskite photoelectrocatalysis has made exceptional progress over the span of a decade, with devices advancing from small laboratory prototypes matching a technology readiness level (TRL) of 3, to scalable outdoor reactors operating for days (TRL 5). Despite perceived drawbacks of perovskite semiconductors, this performance is already comparable with established solar fuel technologies which had much longer to develop, which highlights the potential of these materials moving forward.

While technoeconomic or life cycle analyses can provide valuable estimates, the prices of raw materials, market needs, industrial manufacturing costs or even deposition techniques evolve over time. Working at the crossroads of photoelectrocatalysis and photovoltaics, we often encounter detailed studies that either highlight the potential commercial benefits of photoelectrodes over PV‐electrolysis [112], or vice versa [113]. A recent analysis even indicates that the market value and demand of products may either suit one technology or the other [114]. While the two opposing views are historically hard to reconcile, we instead propose that the best way to prove the potential of a technology is by actually implementing it. Over time, this in itself can decrease prices through economies of scale (see silicon PV prices) and technological improvements (similar to Moore's law for transistors).

To this end, developments in operational stability will need to go hand‐in‐hand with a push toward scalability. Extrapolating from its original definition [115], a TRL of 6 may for instance correspond to a 1 m^2^ reactor, which can successfully produce fuels at a commercially viable STF efficiency of 10% for 1000 h. We expect these technological milestones to be within reach following the earlier described strategies. The insights gained in reactor leakage and stability under variable atmospheric conditions would contribute to designing a modular 100 m^2^ pilot plant (TRL 7), which may draw inspiration from a well‐known example for SrTiO_3_ photocatalysis [95]. This setup would be operated year‐round, providing additional data on roll‐to‐roll batch reproducibility, or gas/liquid product collection and separation, a topic which is often neglected in fundamental (photo)electrocatalytic studies. This platform would provide much more accurate values on operational costs and return on investment, which would enable stakeholders to identify the most promising niches for industrial scale deployment, either on land or at sea (TRL 8–9).

Ultimately, we see lead halide perovskite photoelectrodes as a highly versatile model system, since insights gained in device, catalyst, and reactor design can rapidly translate to other highly processable thin‐film technologies (e.g., third generation PV). Just as developments in organic and dye‐sensitized solar cells acted as a springboard for perovskite photovoltaics [116], we hope that these findings will provide a solid platform for the next best PEC semiconductor to take off.

## 6 Conclusions

Despite their intrinsic moisture degradation, metal halide perovskites have emerged over the past decade as outstanding materials for solar fuel synthesis. While their design may seem complex, it also allows a high degree of control, as band alignment or hydrophobicity can be adjusted to improve performance and stability. As opposed to classical photoelectrodes, a decoupling of light absorption and catalysis also enables greater product selectivity, by taking advantage of the interplay between mass transport, catalyst morphology, local pH effects and current densities. Its compatibility toward new designs also enables perovskite devices to gain new functionality, as demonstrated by the floating artificial leaves or thermoelectrics integration for waste heat harvesting. The introduction of scalable deposition techniques would further facilitate square‐meter prototyping, whereas a push toward the automation of material screening and fabrication would accelerate performance optimization [117]. These efforts could bring perovskite‐based PEC systems gradually from the current technology readiness level of 3–5 (proof‐of‐concept prototypes to small‐scale deployment), toward plant scale prototypes (TRL 6–7), and ultimately to real world applications. This would accomplish the main goal of artificial photosynthesis, by sustaining the high STF performance of complex PV‐electrolysis systems at a much lower balance of cost. Ultimately, such artificial leaves could mimic nature's own carbon cycle, leading to a net‐zero carbon economy.

## Author Contributions

V.A. conceived and wrote the manuscript.

## Conflicts of Interest

The author declares no conflicts of interest.

## Data Availability

Data sharing not applicable to this article as no datasets were generated or analysed during the current study.
